# The effect of pregnancy induced hypertension and multiple pregnancies on preterm birth in Ethiopia: a systematic review and meta-analysis

**DOI:** 10.1186/s13104-019-4128-0

**Published:** 2019-02-18

**Authors:** Getaneh Mulualem, Amare Wondim, Abere Woretaw

**Affiliations:** 1 Department of Pediatrics and Child Health Nursing, School of Nursing, College of Medicine and Health Sciences, University of Gondar, P.O.BOX: 196, Gondar, Ethiopia; 2 Department of Medical Nursing, School of Nursing, College of Medicine and Health Sciences, University of Gondar, P.O.BOX: 196, Gondar, Ethiopia

**Keywords:** Ethiopia, Multiple pregnancy, Pregnancy induced hypertension, Preterm birth

## Abstract

**Objective:**

This systematic review and meta-analysis aimed to estimate the overall pooled prevalence of preterm birth and the effect of pregnancy induced hypertension (PIH) and multiple pregnancies on preterm birth in Ethiopia.

**Result:**

A total of 9 studies with 27,119 participants were included in this systematic review and meta-analysis. The pooled prevalence of preterm birth among mothers who gave births in Ethiopia was found to be 13.32% (95% CI = 7.99, 18.660). Preterm birth was found to be higher among mothers who had pregnancy induced hypertension with odds ratio of 4.69 (95% CI = 2.32, 9.49) and multiple pregnancy with odds ratio of 2.40 (95% CI = 1.06, 5.45) as compared to the counterparts. In subgroup analysis by region, the prevalence of preterm birth was found to be 12.63% (95% CI = 3.26, 22) in Amhara and 10.18% (95% CI = 6.04, 14.32) in Oromia region.

**Electronic supplementary material:**

The online version of this article (10.1186/s13104-019-4128-0) contains supplementary material, which is available to authorized users.

## Introduction

Preterm birth is defined as babies born alive before 37 weeks of pregnancy is completed. Globally, every year, an estimated 15 million babies are born before 37 weeks of gestation of which 85% of the preterm births are concentrated in Africa and Asia [1, 2]. Preterm birth is one of the major determinants of neonatal morbidity and mortality [3]. Worldwide, 27% of the direct leading cause of neonatal death is preterm birth; more than one million preterm newborns die annually [4].

Developmental immaturity affects a wide range of organ systems in preterm neonates. Preterm birth results in short term complications, such as respiratory distress syndrome, apnea and brain immaturity [5]. Many of them develop lifelong complications, like pneumonia, respiratory failure, cerebral palsy, neurological impairment, mental retardation, visual and hearing impairments and poor health [6–10].

Around 75% of the preterm births result from spontaneous preterm labour. The remaining 25% are delivered for medical reasons [11]. The causes of preterm birth are multifactorial [12]. These may be history of pregnancy induced hypertension [13–17], premature rupture of membrane [14, 15, 17], placenta previa, abruption placenta [16], maternal depression symptoms [18], previous indicated preterm birth [13, 17], lung diseases, heavy work during pregnancy [13], multiple gestations [14, 16], urinary tract infections [15, 16], history of stillbirth, history of miscarriage [16], and inadequate antenatal visits [16, 17].

Use of alternative models of antenatal care [19], treating intra-uterine infections, improving maternal nutrition, maternal lifestyle modification [20], smoking cessation, reduction of non-medically indicated labour induction or caesarean delivery are common strategies used to reduce preterm birth [8]. In Ethiopia, even though there is a strong initiative in antenatal care service implementations [21], different studies show the prevalence of preterm birth. Therefore, this systematic review and meta-analysis will determine the effect of PIH and multiple pregnancies on preterm birth in Ethiopia.

## Main text

### Methods

#### Protocol and registration

This protocol has been registered with the International Prospective Register of Systematic Reviews (PROSPERO). The web address and the registration number of this systematic review and meta-analysis are https://www.crd.york.ac.uk/PROSPERO and CRD42019118389 respectively.

#### Reporting

The preferred reporting items of systematic reviews and meta-analysis (PRISMA) guideline [22] was used to report the result of this study (Additional file 1).

#### Inclusion and exclusion criteria

In this study, articles which fulfilled the criteria, (1) observational studies including cohort, cross-sectional, and case–control studies; (2) articles that report the prevalence of preterm birth and/studies that report the association of pregnancy induced hypertension (PIH) and/multiple pregnancy) with preterm birth; (3) studies conducted in Ethiopia; (4) published and unpublished articles at any time; (5) studies which have been written by English, and (6) Studies conducted both at community or institution level were included. Conference papers, editorials, articles without full texts, trials, systematic reviews and meta-analyses, and qualitative studies were excluded.

#### Outcome measurement

Preterm birth is defined as babies born alive before 37 weeks of pregnancy is completed [2].

#### Databases and searching strategy

We searched all available articles with electronic databases including, PubMed, EMBASE, web of science, and Google scholar. Additionally, we searched using the reference list of included studies and the Ethiopian institutional research repository. Articles were searched using the following search terms: “preterm”, “low gestational age”, “preterm birth”, “preterm delivery”, “early delivery”, “pregnancy induced hypertension”, “hypertension”, “preeclampsia”, “eclampsia”, “pregnancy”, “multiple pregnancy”, “twin pregnancy”, “prevalence”, “incidence”, “predictors”, “factors”, “risk factors”, and “Ethiopia”. A searching string was developed using “AND” and “OR” BOOLEN operators. PubMed searching was done using this searching string (Additional file 2).

#### Study selection and quality assessment

First, all identified studies were imported to Endnote 7 citation manager. Second, duplicates were carefully removed. Third, two independent authors (GM and AW) were screened and assessed the title and abstracts followed by full text assessment. Any disagreements between authors were solved by discussion and consensus. Fourth, Two investigators (GM and AW) assessed the quality of studies using the JBI quality appraisal criteria [23]. Any disagreements were solved by discussion and repeating the procedures. For assessing the quality, we used the JBI critical appraisal checklist adapted for cross-sectional, case–control, and cohort studies (Additional file 3) [23]. Studies considered low risk whenever fitted to 50% and or above quality assessment checklist criteria’s.

#### Data extraction

After quality assessment, two independent authors (GM and AW) extracted the data in excel Microsoft spreadsheet. The extracted data items were, first author, year of publication, study area, region, design, population, sample size, prevalence of preterm birth, OR of PIH, and multiple pregnancies. Any disagreements were solved by discussion.

#### Data analysis

A weighted inverse variance random- effects model [24] was used. Subgroup analysis by region was done to estimate regional variations in the prevalence of preterm birth. The percentage of total variation between studies due to heterogeneity was assessed with I^2^ [25]. I^2^ test statistics results of 25%, 50%, and 75% were declared as low, moderate, and high heterogeneity, respectively [25]. Publication bias was assessed by funnel plot and Egger’s test. Statistically significant publication bias was declared at P-value less than 0.05. STATA version 11 statistical software was used for statistical analysis.

### Result

#### Search results

**Fig. 1:**
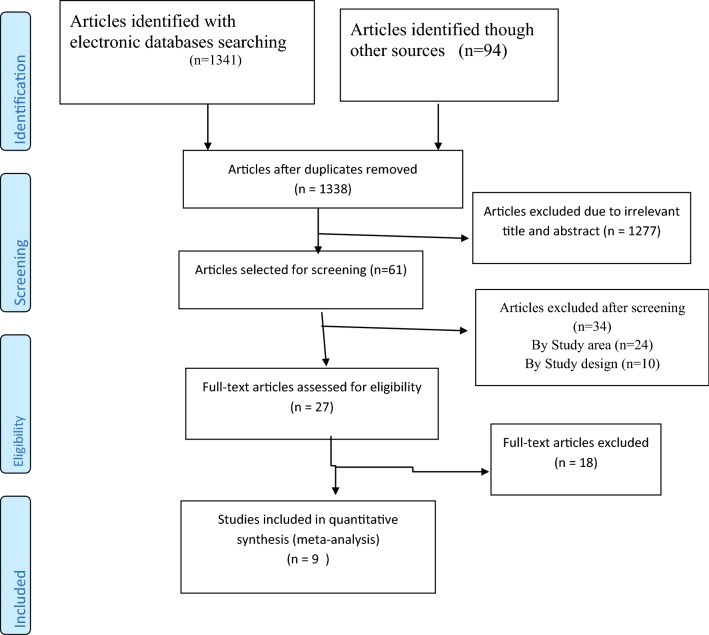
A PRISMA flow diagram of articles screening and process of selection

On the whole, we searched 1435 articles from different data sources of which 1307 articles were from PubMed, 71 from Google scholar, 23 from EMBASE, 11 from web of science, 3 from reference lists of included studies, 7 from institutional research repositories, and 13 from Google. Ninety-seven articles were removed due to duplicates, 1277 due to irrelevant titles and abstracts, 24 due to study area (done in another country), and 10 due to study design. Twenty-seven articles were selected for the full text review of which 18 were excluded after the full text review. Finally, 9 studies were included in this systematic review and meta-analysis to estimate the effect of PIH and multiple pregnancies on preterm birth and pooled prevalence of preterm birth in Ethiopia (Fig. 1).

#### Characteristics of included studies

A total of 9 studies with 27, 119 participants were included in this systematic review and meta-analysis. Out of nine studies conducted, three [26–28] were from Amhara region, three [29–31] from Tigray, two [17, 32] from Oromia, and one [33] from Addis Abeba. Regarding the study design, 6 studies [26–28, 31–33] were cross-sectional, two [17, 30] case–control, and one [33] retrospective follow up. The highest prevalence of preterm birth was reported from Oromia (25.9%) [32] and the lowest from Amhara region (4.4%) [27] (Table 1).

**Table 1:** General characteristics of included studies that report the prevalence of preterm and/its association with pregnancy induced hypertension and multiple pregnancy

Author/year of publication	Study area	Region	Study design	Study population	Sample size	Prevalence (%)	Result of quality
Mengesha et al./2016 [29]	Hospital	Tigray	Cohort	Mothers gave birth	1152	6	Low risk
Gebresillasie/2016 [27]	Gondar Town	Amhara	Cross-sectional	Mothers gave birth	540	4.4	Low risk
Deressa et al./2018 [33]	Addis Abeba Hospital	Addis Abeba	Cross sectional	Mothers gave birth	23,115	16.5	Low risk
Bekele et al./2015 [26]	Debremarkos Town	Amhara	Cross sectional	Mothers gave birth	422	11.6	Low risk
Adhena et al./2017 [31]	Shirie Suhul Hospital	Tigray	Cross sectional	Mothers gave birth	425	8.7	Low risk
Sakata et al./2017 [28]	Gondar University Hospital	Amhara	Cross sectional	Mothers gave birth	325	22.5	Low risk
Bekele et al./2017 [32]	Jimma Hospital	Oromia	Cross sectional	Mothers gave birth	220	25.9	Low risk
Abaraya et al./2018 [17]	Jimma University Hospital	Oromia	Case control	Mothers gave birth	656	NA	Low risk
Teklay et al./2017/2018 [30]	Mekelle Hospital	Tigray	Case control	Mothers gave birth	264	NA	Low risk

#### Quality of included studies

Out of nine studies, six were assessed with JBI critical appraisal checklist for cross-sectional studies, two studies with a JBI critical appraisal for case–control, and one study with JBI checklist for cohort studies. None of the studies were excluded after quality assessment. The results were described in (Table 1).

#### Meta-analysis

A significant publication bias was not observed in this study. Hence, visual inspection of funnel plot was symmetrical, and Egger’s regression P-value was 0.303 (Additional file 4).

#### Prevalence of preterm birth

**Fig. 2:**
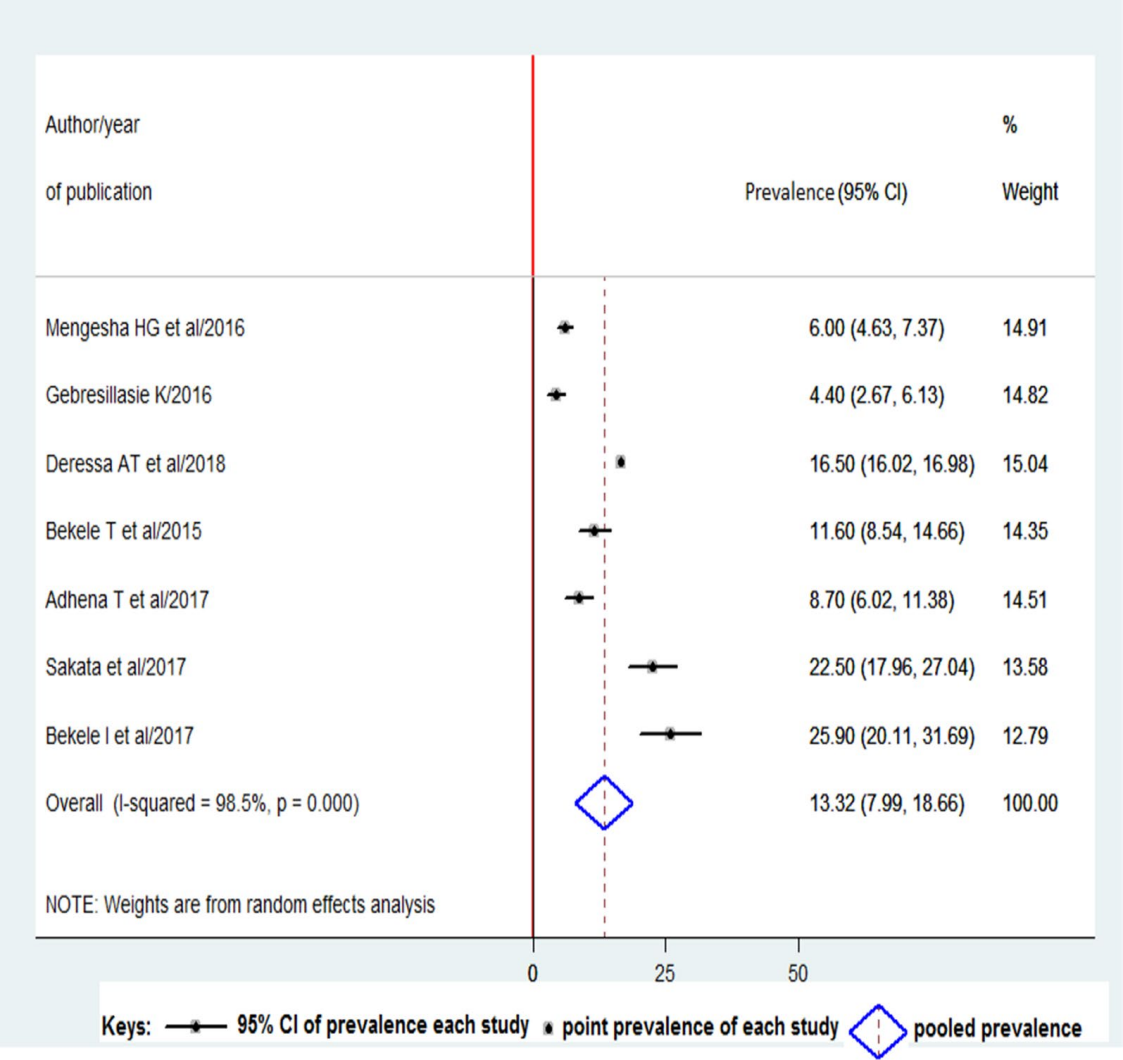
Forest plot of the prevalence of preterm birth with 95% Cl. The midpoint and length of each segment indicated the prevalence and 95% confidence interval. The diamond shape revealed the pooled prevalence

Out of 9 studies, seven [26–29, 31–33] reported the prevalence of preterm birth considered in the meta-analysis to estimate the pooled prevalence of preterm birth in Ethiopia. The pooled prevalence of preterm birth in Ethiopia was found to be 13.32% (95% CI = 7.99, 18.66; I^2^ = 98.5%; P = 0.000) (Fig. 2). Egger’s regression test P-value = 0.303.

We performed a subgroup analysis by region. Consequently, the pooled prevalence by region was 12.63% (95% CI = 3.26, 22; I^2^ = 96.7%; P = 0.000) for Amhara and 10.18% (95% CI = 6.04, 14.32; I^2^ = 93.9%; P = 0.000) for Oromia (Additional file 5).

#### The association of PIH and multiple pregnancies with preterm birth

To further explore the association between PIH and preterm birth, five studies [17, 27, 30, 32, 33] reported extractable data on preterm birth among mothers with PIH and with no PIH. One study [33] is excessively influential, and not considered in the meta-analysis. In the current study, preterm birth was 4.69 times higher 4.69 (95% CI = 2.32, 9.49; I^2^ = 67.6%; P = 0.026) among mothers who had pregnancy induced hypertension compared to mothers with no PIH (Additional file 6). Moreover, the association between preterm birth and multiple pregnancy was explored. Out of 9 studies, five [17, 28, 30, 32, 33] reported sufficient data to calculate the odds ratio. Consequently, preterm birth among multiple pregnancies was found to be 2.4 times higher 2.40 (95% CI = 1.06, 5.45; I^2^ = 85.6%; P = 0.000) compared to single pregnancy (Additional file 7).

### Discussion

Despite the advancement of preterm birth management, the proportion of preterm birth and its being a major and direct cause of neonatal mortality is still high among delivering mothers [34]. So far, there is no previous systematic reviews/meta-analyses that have examined the national estimate of preterm birth in Ethiopia. The aim of this systemic review and meta-analysis was to assess the effect of pregnancy induced hypertension and multiple pregnancy on preterm birth in the country.

Although Ethiopia was not mentioned among the top ten countries with the highest rates of preterm birth per 100 live births in the world, inconsistency of findings was reported with regard to the problem. The overall pooled prevalence of preterm birth on this study was found to be 13.32 (95% CI 7.99, 18.66). This finding is lower than that of Kenya (20.2%) [35] and (18.3%) [14], 18.01% in India [36], 14.2% in Tanzania [16], 16.81% in Pakistan [37] but higher than 12.8% in Jordan [38], 12.3% in Brazil [39], and 9.2% in Iran [40]. The high prevalence of preterm birth in Ethiopia could be due to risk factors, like the occurrence of infections during pregnancy, several lifestyle conditions (stress, strenuous work, standing work), short inter-pregnancy interval, and low body mass index thought to be high although half of the preterm births occur idiopathically [41].

Subgroup analysis revealed that there was a significant variation among regions. Preterm birth in Amhara region was higher than that of Tigray. The difference might be because mothers in Tigray had health setups, infrastructures, and better accesses to healthcare settings that have the potential to reduce preterm birth than mothers in Amhara.

Concerning the predictors, enormous risks, including demographic, social, and medical were identified [14, 16, 39, 42]. Among the risks PIH was in increasing trend [43]. In this study, mothers with PIH were nearly 4.7 times more likely to give preterm births than those who had no hypertension (OR = 4.69; 95% CI = 2.32, 9.49). This finding is in line with those of studies conducted in Tanzania [16], Kenya [35], and report from the United Kingdom [44]. This might be due to the fact that PIH could cause vascular damage to the placenta, which induces the oxytocin receptors, resulting in preterm labor and delivery. Besides, hypertension decreases the utero-placental blood floor, which leads to intrauterine growth restriction that causes preterm delivery.

The odds of a mother with multiple pregnancies were 2.4 times more likely to give preterm birth than mothers with single pregnancies (OR = 2.40; 95% CI 1.06, 5.45). The finding is supported by those of cohort studies, and systematic reviews in China [45], Tanzania [16], and Kenyatta National hospital, Kenya [14]. This is probably due to the over distension of the uterus by multiple pregnancies which stimulate early labour leading to preterm delivery. Moreover, other complications, like pre-eclampsia, and polyhydramnios are more likely to occur with multiple gestations and contribute to iatrogenic preterm birth.

### Conclusion

Preterm birth in Ethiopia is a significant problem. Though the determinants of preterm birth are multifactorial, PIH and multiple pregnancies remained a major contributing factor to the risk of preterm birth. Preconception counseling and antenatal care to facilitate timely management of PIH, more specially controlling hypertension is very important to maintain blood flow to the fetus. Improving health care quality delivered to pregnant women may reduce risk factors for preterm delivery.

## Limitations

Strength of this review: Since there has been no similar previous study, this review and meta-analysis showed the national pooled image of preterm birth in Ethiopia and the effect of pregnancy induced hypertension and multiple pregnancy on preterm birth. Strictly following PRISMA guide line and Joanna Briggs Institute Meta-Analysis of Statistics Assessment and Review Instrument (JBI-MAStARI) during critical appraisal is the additional strength of this systemic review and meta-analysis.

The search strategy was limited to articles published in English, and this could lead to reporting bias. Relevant predictors might have been missed; hence, future reviews should consider other factors of preterm birth to explore the inquiry more deeply on subject of inquiry. Furthermore, presence of high statistical heterogeneity among studies conducted on preterm birth were considered as limitation of this review.

## Additional files

**Additional file 1.** PRISMA checklist.

**Additional file 2.** Searching strings used for PubMed.

**Additional file 3.** JBI critical appraisal checklist for cross-sectional, case–control and cohort studies.

**Additional file 4: Fig. S1.** Funnel plot for publication bias, log p or LNP (log of proportion in the X-axis and standard error of log proportion in the Y-axis.

**Additional file 5: Fig. S2.** Forest plot of subgroup analysis of the prevalence of preterm birth by region with 95% CI. The midpoint and the length of each segment revealed the prevalence and CI. The diamond shape showed combined prevalence.

**Additional file 6: Fig. S3.** Forest plot of OR of preterm birth among mothers with PIH. The midpoint and the length of each segment indicated OR and 95% CI respectively. The diamond shape indicated the pooled OR.

**Additional file 7: Fig. S4.** Forest plot of OR of preterm birth among multiple pregnancies with 95% CI. The midpoint and the length of each segment indicated OR and 95% CI respectively. The diamond shape indicated the pooled OR.

## Abbreviations

CI: confidence interval
JBI: Joanna Briggs Institute
OR: odds ratio
PIH: pregnancy induced hypertension
