# Supplementary figures and images for: The effect of pregnancy induced hypertension and multiple pregnancies on preterm birth in Ethiopia: a systematic review and meta-analysis
Source: BMC Res Notes. 2019 Feb 18;12:91. doi: 10.1186/s13104-019-4128-0 (PMC6380048; doi:10.1186/s13104-019-4128-0)

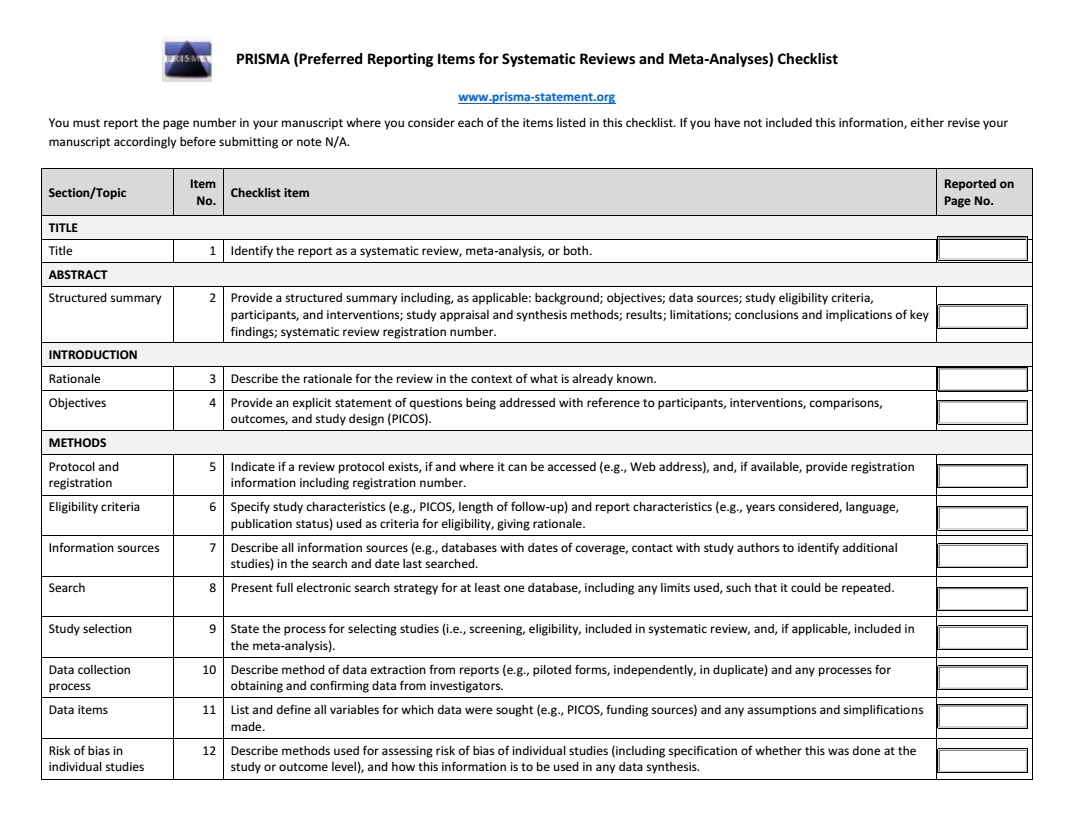


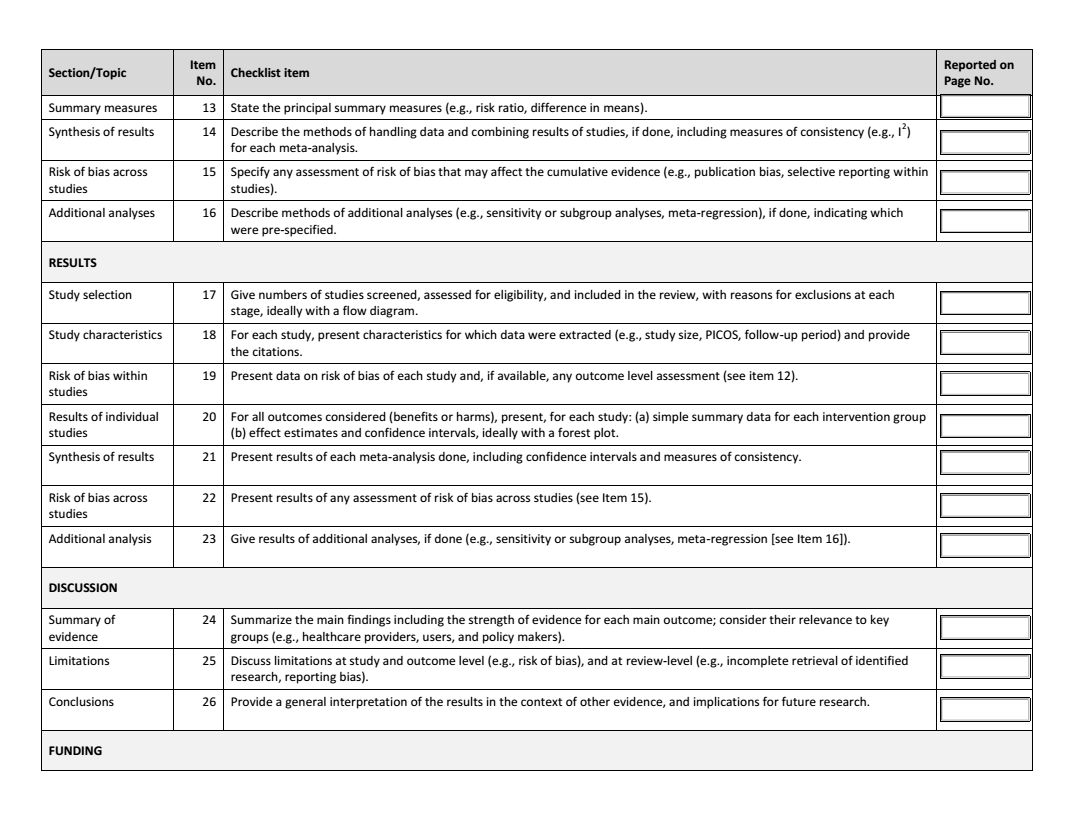

Supplement: Supplementary file 1 — Additional file 1. PRISMA checklist. [file 13104_2019_4128_MOESM1_ESM.docx]

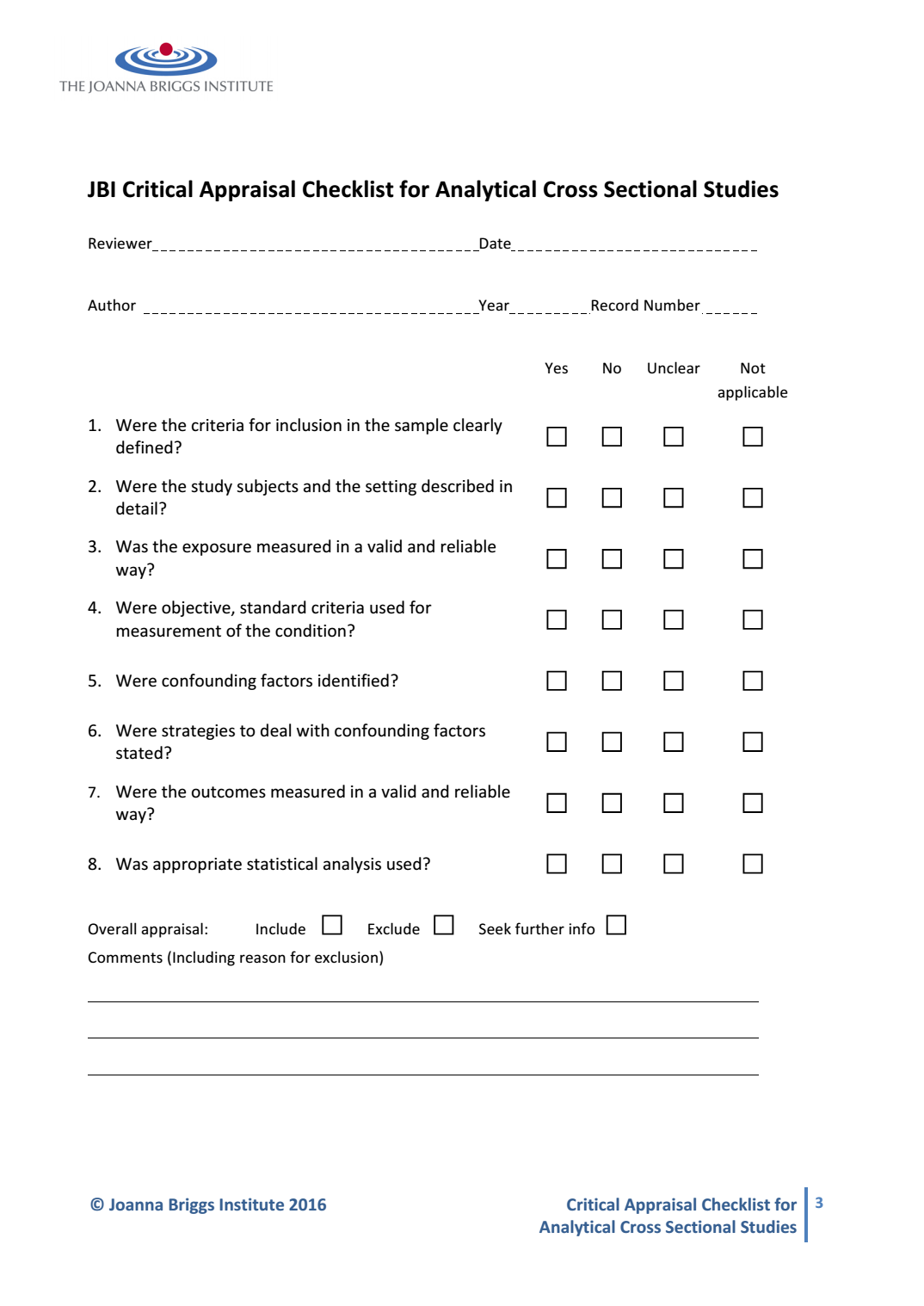


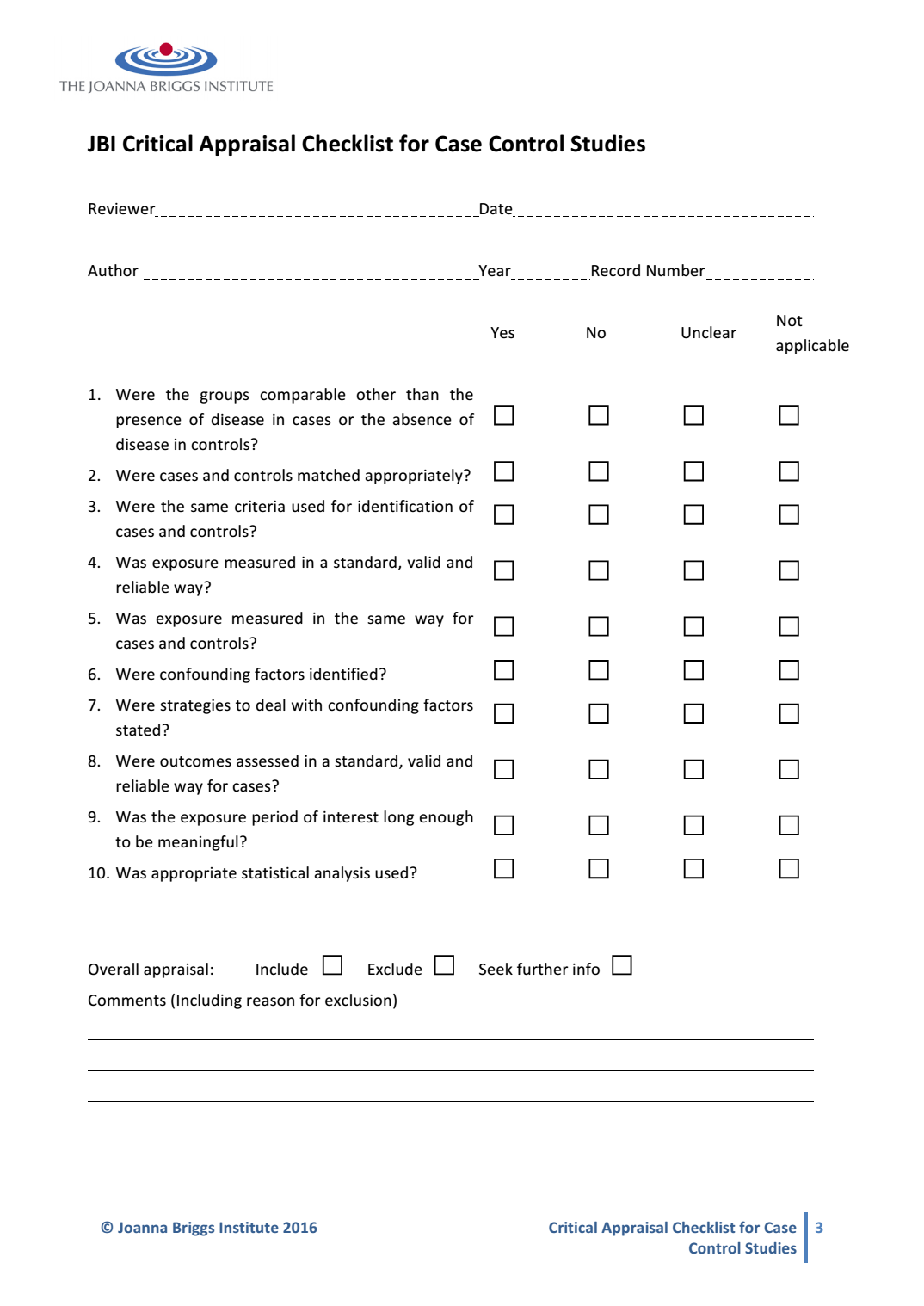


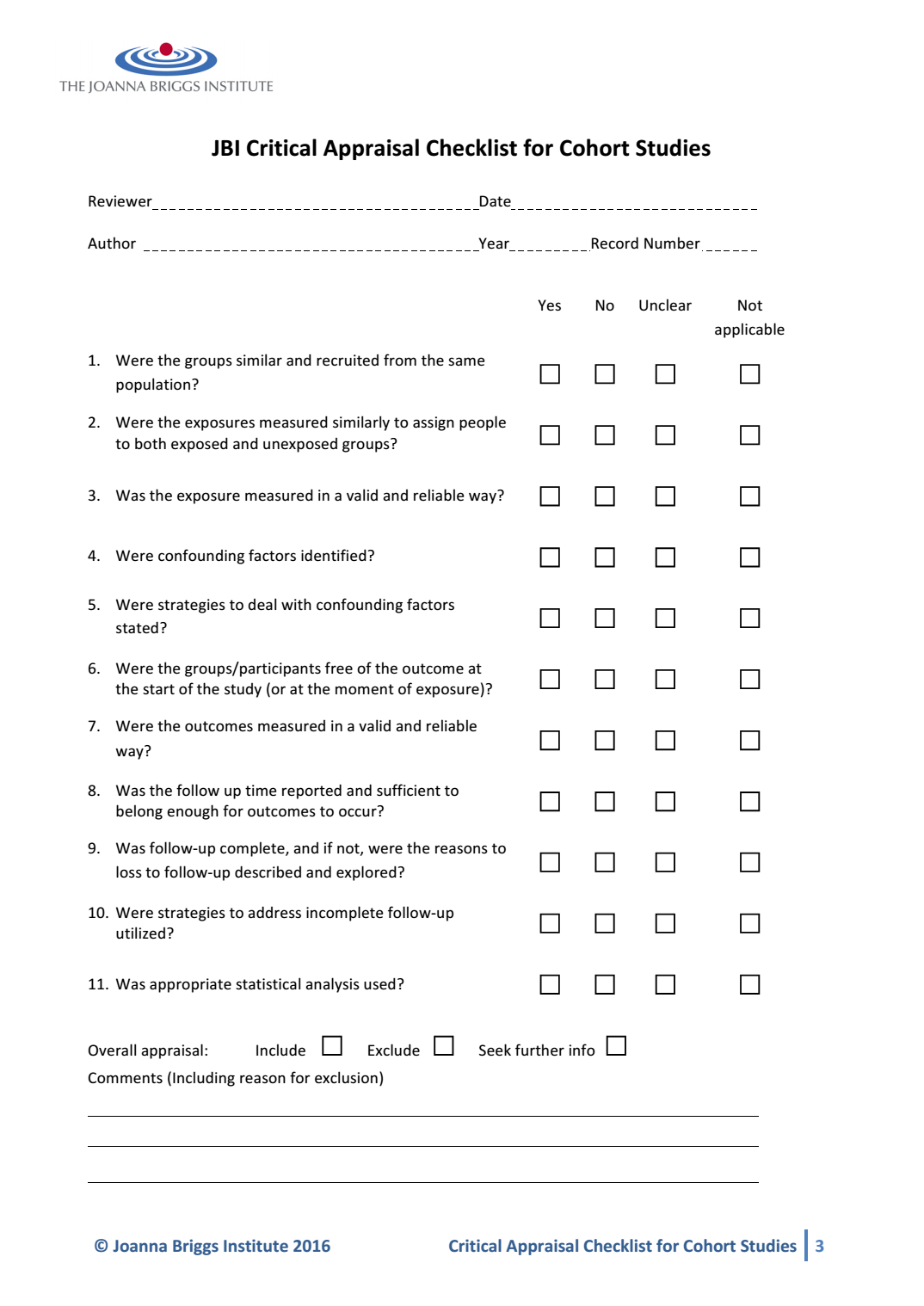

Supplement: Supplementary file 3 — Additional file 3. JBI critical appraisal checklist for cross-sectional, case–control and cohort studies. [file 13104_2019_4128_MOESM3_ESM.docx]

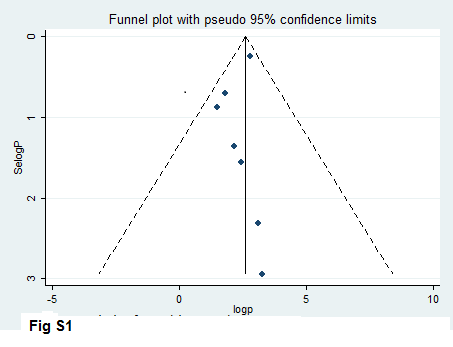

Supplement: Supplementary file 4 — Additional file 4: Fig. S1. Funnel plot for publication bias, log p or LNP (log of proportion in the X-axis and standard error of log proportion in the Y-axis. [file 13104_2019_4128_MOESM4_ESM.docx]

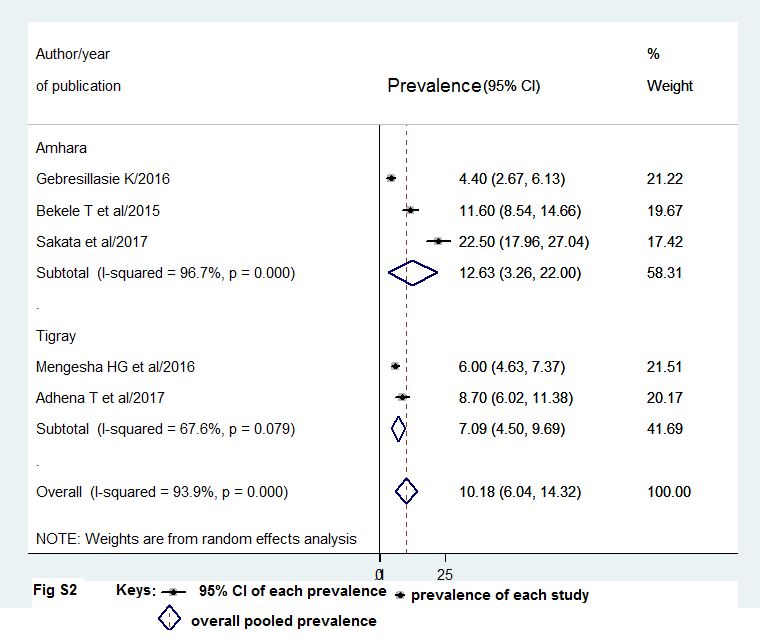

Supplement: Supplementary file 5 — Additional file 5: Fig. S2. Forest plot of subgroup analysis of the prevalence of preterm birth by region with 95% CI. The midpoint and the length of each segment revealed the prevalence and CI. The diamond shape showed combined prevalence. [file 13104_2019_4128_MOESM5_ESM.docx]

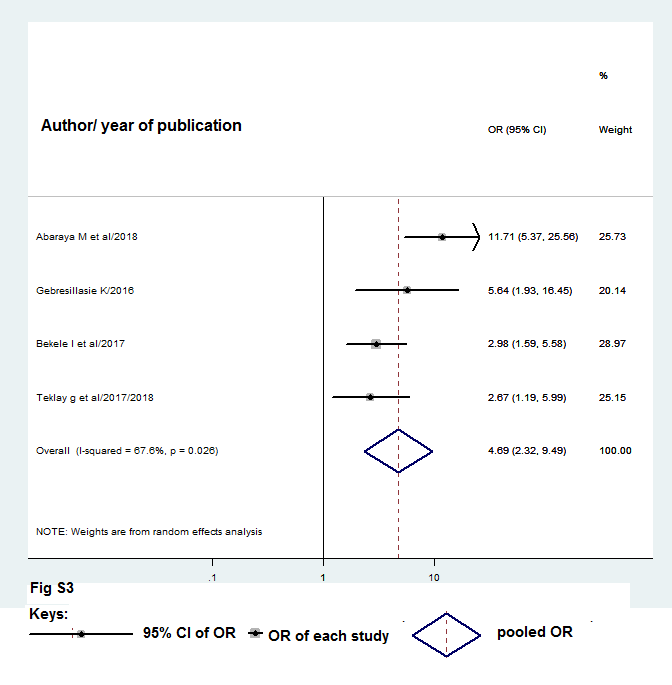

Supplement: Supplementary file 6 — Additional file 6: Fig. S3. Forest plot of OR of preterm birth among mothers with PIH. The midpoint and the length of each segment indicated OR and 95% CI respectively. The diamond shape indicated the pooled OR. [file 13104_2019_4128_MOESM6_ESM.docx]

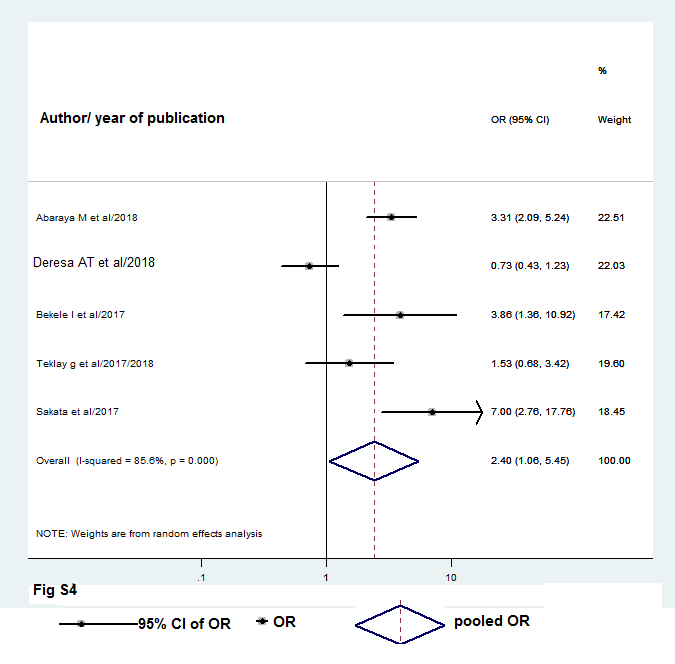

Supplement: Supplementary file 7 — Additional file 7: Fig. S4. Forest plot of OR of preterm birth among multiple pregnancies with 95% CI. The midpoint and the length of each segment indicated OR and 95% CI respectively. The diamond shape indicated the pooled OR. [file 13104_2019_4128_MOESM7_ESM.docx]
