# Supplementary material for: The effect of pregnancy induced hypertension and multiple pregnancies on preterm birth in Ethiopia: a systematic review and meta-analysis
Source: BMC Res Notes. 2019 Feb 18;12:91. doi: 10.1186/s13104-019-4128-0 (PMC6380048; doi:10.1186/s13104-019-4128-0)
Supplement: Supplementary file 2 — Additional file 2. Searching strings used for PubMed. [file 13104_2019_4128_MOESM2_ESM.docx]

((effect) OR (impact[MeSH Terms]) AND (pregnancy induced hypertension) OR (PIH[MeSH Terms]) OR (preeclampsia) OR (eclampsia[MeSH Terms]) AND (multiple pregnancy) OR (twin pregnancy[MeSH Terms]) AND (prevalence) OR (incidence[MeSH Terms]) AND (preterm birth) OR (preterm delivery[MeSH Terms]) OR (low gestational age delivery) AND (associated factors) OR (predictors[MeSH Terms]) OR (risk factors[MeSH Terms]) AND (Ethiopia))
